# Snow White and the Seven Dwarfs: a fairytale for endocrinologists

**DOI:** 10.1530/EC-20-0615

**Published:** 2021-04-16

**Authors:** Athanasios Zervas, George Chrousos, Sarantis Livadas

**Affiliations:** 1 Endocrine Unit, Athens Medical Centre, Athens, Greece; 2 Division of Endocrinology, Metabolism and Diabetes, First Department of Pediatrics, University Research Institute of Maternal and Child Health and Precision Medicine, and UNESCO Chair on Adolescent Health Care, National and Kapodistrian University of Athens, Athens, Greece; 3 National and Kapodistrian University of Athens Medical School, ‘Aghia Sophia’ Children’s Hospital, Athens, Greece

**Keywords:** growth, dwarfism, pituitary, pseudohypoparathyroidism

## Abstract

‘Snow White and the Seven Dwarfs’, a fairytale that is widely known across the Western world, was originally written by the *Brothers Grimm,* and published in 1812 as ‘Snow White’. Though each dwarf was first given an individual name in the 1912 Broadway play, in *Walt Disney*’s 1937 film ‘Snow White and the Seven Dwarfs’*,* they were renamed, and the dwarfs have become household names. It is well known that myths, fables, and fairytales, though appearing to be merely children’s tales about fictional magical beings and places, have, more often than not, originated from real facts. Therefore, the presence of the seven brothers with short stature in the story is, from an endocrinological point of view, highly intriguing, in fact, thrilling. The diversity of the phenotypes among the seven dwarfs is also stimulating, although puzzling. We undertook a differential diagnosis of their common underlying disorder based on the original Disney production’s drawings and the unique characteristics of these little gentlemen, while we additionally evaluated several causes of short stature and, focusing on endocrine disorders that could lead to these clinical features among siblings, we have, we believe, been able to reveal the underlying disease depicted in this archetypal tale.

## Introduction

The famous Brothers Grimm fairytale ‘Snow White’ has been, without a doubt, an enchanting narrative for every Western child during at least the past two centuries. Meanwhile, for the endocrinologist, the folktale portrayal of seven dwarf brothers, very possibly based on an early representation of certain facts, constitutes a difficult diagnostic challenge. The aim of this paper was to discover and as closely as possible attribute a distinctive clinical condition to each of the dwarf male members of the same family and to provide the reader with a scientific or clinical explanation of the diversity of phenotypes described by the Brothers Grimm and depicted in Walt Disney’s 1937 film ‘Snow White and the Seven Dwarfs’.

The art of the differential diagnosis is based on the combination of a detailed clinical examination and assembly of a patient’s personal and family history, augmented by the physician’s scientific knowledge and experience, which will guide them through appropriate laboratory testing to the correct diagnosis (1). However, as in this case, no laboratory tests can be available for evaluation, our diagnosis must be based on the clinical characteristics of the dwarfs, as they were set down by Disney’s illustrators and shown on celluloid.

**Figure 1:**
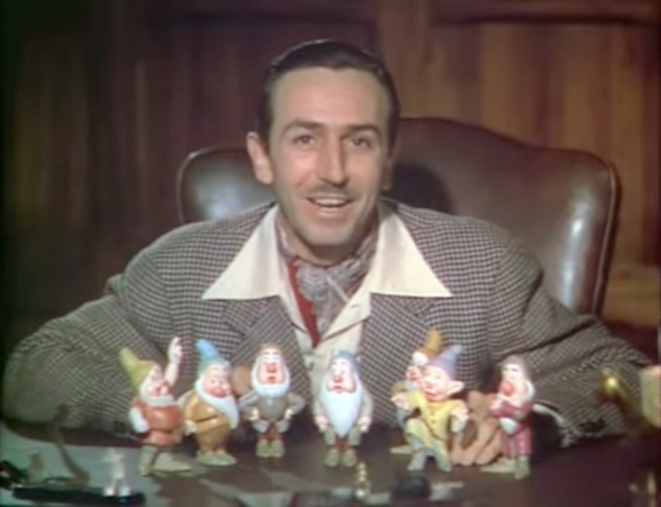
Walt Disney presenting the dwarfs’ characteristics in the official theatrical trailer for *Snow White and the Seven Dwarfs* (1937). Walt Disney, Public domain, via Wikimedia Commons (https://commons.wikimedia.org/wiki/File:Snow_White_and_the_Seven_Dwarfs_trailer_(1937).webm).

**Figure 2:**
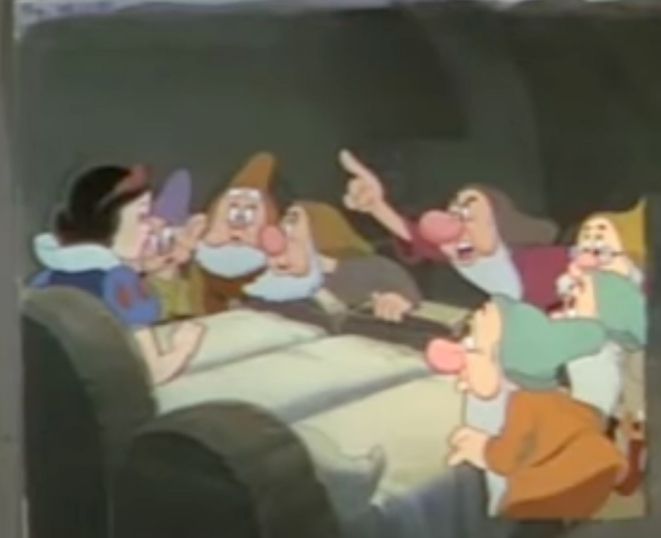
The dwarfs with Snow White in the official theatrical trailer for *Snow White and the Seven Dwarfs* (1937). Walt Disney, Public domain, via Wikimedia Commons (https://commons.wikimedia.org/wiki/File:Snow_White_and_the_Seven_Dwarfs_trailer_(1937).webm).

In the 1937 Broadway production recounting the story, the seven dwarfs were first given individual names (their names and personalities are not stated in the original fairytale); Walt Disney not only furnished them with different names but also elaborated upon their individual characteristics (2) (Figs 1 and  2). Judging from the attributes he gave them, it is very likely that Walt Disney’s illustrators were familiar with a family of dwarfs, granted that their movie features are, indeed, typically encountered in subjects with dwarfism. The dwarfs’ names were chosen from a pool of about 50 potential ones, with the final names adapted being Doc, Grumpy, Happy, Sleepy, Bashful, Sneezy, and Dopey. These names and the characteristics of each dwarf are listed in Table 1 (3). In our study, the original Disney film production drawings (4) were used in an attempt to determine the seven dwarfs’ phenotypes, which was critical in pursuing our clinical investigation of these well-known characters.

**Table 1:** Names and characteristics of the seven dwarfs.

Name	Characteristics
Doc	He is the leader of the seven dwarfs, wears glasses, and often mixes up his words.
Bashful	Very shy and sweet. He is also described as cute. He also has a rosy, blushy face and a small shy smile.
Grumpy	He has the biggest nose of the dwarfs and is frequently seen with one eye shut.
Sneezy	Sneezy’s name is due to his extraordinarily powerful sneezes (caused by hay fever), which are seen blowing even the heaviest of objects across a room.
Sleepy	Sleepy is always tired and appears slothful in most situations.
Happy	Happy is the joyous dwarf and is usually portrayed laughing.
Dopey	Dopey is the only dwarf who does not have a beard. He is clumsy and mute.

## How short were our heroes?

Any modern-day movie dealing with a crime or crimes typically shows the scene where the arrested individual has a ‘mug shot’ taken of him at the police station – via which we may also gain a rough idea of his height or stature. Definitely, in any enquiry into an individual’s characteristics, height is of key importance. Thus, our first query concerns the actual stature of our heroes. The original production drawings (4) include the following specifics: ‘Doc is about ¼ head taller than the other dwarfs’*,* ‘Grumpy is the average size of dwarfs’*,* and ‘Dopey is a little shorter than the [others]’. In the same drawings, we see horizontal lines showing the comparative sizes of the dwarfs and Snow White. Based on the latter, we estimated that the height ratio between Snow White and the dwarfs ranges from 1.77 (Doc) to 1.95 (Dopey). Grumpy was, thus, of average size among the dwarfs, with a ratio of 1.88.

Accordingly, the next question was: what was Snow White’s height? Judging from the facial traits in the production drawings and from the story itself (e.g. Love’s First Kiss given by the Prince), we may assume that Snow White was 15–17 years old, that is, in mid-to-late adolescence. At this age, according to the Centers for Disease Control and Prevention (CDC) growth charts, the mean height of the American female ranges from 161.1 to 162.5 cm (5). Given that the mean female height has not changed substantially during the last 100 years (6) and assuming that Disney wanted a body composition familiar to the average filmgoer, we conclude that the abovementioned range may represent Snow White’s actual height. Thus, the dwarfs’ heights should range between 82.56 and 83.28 cm (Dopey) to between 90.61 and 91.40 cm (Doc), with the rest of the dwarfs distributed in between. Short stature (SS) is defined as a height below the third percentile or greater than two s.d. below the mean height for chronological age. Hence, if our hypothesis is correct, the dwarfs’ height will certainly have been collectively below the fourth centile on the CDC growth chart (7). According to the current UK guidelines, this is a height compatible with the diagnosis of dwarfism, which considers evaluation for short stature after a single height measurement lower than the fourth centile at 5 years of age (the Coventry Consensus) (8).

## The unusual suspects

The second query was what kind of dwarfism our little fellows suffered from. According to one of the currently used classifications, the causes of short stature are numerous (Table 2), and the list is constantly growing (9). Indeed, over the last three decades, extraordinary advances in our understanding of the genetic bases of growth abnormalities have taken place, with the identification of many monogenic causes of growth disorders. In addition, genome-wide association studies have revealed more than 600 variants associated with several human traits, explaining small fractions of phenotypic variations. It was determined that height is a classic polygenic trait that justifies approximately 80% of the variance (10, 11).

**Table 2:** Classification of short stature.

ISS of known etiology
(A) Disproportionate
(1) Congenital: skeletal dysplasias
(2) Acquired: secondary to malformations, radiotherapy, tumors, and other diseases
(B) Proportionate of prenatal origin (newborn SGA)
Due to fetal factors
• Chromosomopathies (Turner, Down, Prader–Willi, etc.) • Syndromic (Silver–Russell, Cornelia de Lange, Noonan, etc.) •Primordial dwarfism (MOPD I, II, III)
Due to uterine of placental features
Due to maternal features
• Malnutrition • Drugs • Cardiac pathology • Congenital infections (TORCH)
(C) Proportionate of postnatal origin
Malnutrition
Chronic infectious diseases
Organic diseases
• Gastrointestinal (celiac disease, Crohn’s disease, cystic fibrosis, short intestine, etc.) • Hepatic (biliary atresia, chronic hepatitis, liver transplantation, etc.) • Renal (glomerular, interstitial, tubular) • Cardiac (cianosant congenital cardiopathies) • Pulmonary (asthma, bronchopulmonary dysplasia, obstructive apnea, etc.) • Metabolic (poorly controlled diabetes mellitus) • Hematological (chronic severe anemia, hemochromatosis) • Oncological (leukemias, lymphomas, tumors of the CNS, etc.) • CNS (idiopathic cerebral palsy, mental retardation, etc.) • Rheumatological (chronic juvenile arthritis, systemic lupus erythematosus, etc.)
Endocrine diseases
• Growth hormone/IGF-1 deficiency or insensitivity • Hypothyroidism • Hypercortisolism • Precocious puberty • Pseudohypoparathyroidism • Inherited rickets (hypocalcemic and hypophosphatemic) • Diabetes mellitus with poor control • Diabetes insipidus without treatment
Psychosocial
II SS of unknown etiology: ISS
Normal variants of SS
FSS • CDGP • Association of FSS and CDGP
Other causes as yet unknown

CDGP, constitutional delay of growth and puberty; FSS, familial SS; ISS, idiopathic SS, SS, short stature.

The initial step in the evaluation of a subject with short stature is to determine whether it is proportionate or disproportionate and whether any dysmorphic features exist. Proportionate short stature defines an unusually small person, whose body parts are appropriate for the age, whereas in disproportionate short stature, the limbs are small compared with the trunk (12, 13). As seen in the drawings, none of the dwarfs exhibited disproportionality between trunk and limbs, which excludes congenital and acquired causes of disproportionate short stature. Therefore, disorders, such as the mucopolysaccharidoses, osteogenesis imperfecta, dyschondrosteoses, mucolipidoses, achondroplasia, hypochondroplasia, Leri–WeiIl dyschondrosteosis, and hypophosphatemic rickets should be excluded.

With regard to the issue of dysmorphic features, there are a number of signs that lead to the diagnosis of several syndromes, for example, the existence of a short nose with anteverted nostrils (Smith–Lemli–Opitz), continuous eyebrows (Cornelia de Lange), the absence of adipose tissue (leprechaunism), or alopecia (progeria) (14). However, based on the production drawings and on the characters as seen in the movie, we observe no significant characteristics pointing to any of the various syndromes associated with dysmorphic features, such as chromosomopathies, and syndromic or primordial dwarfism.

We, thus, concluded that our heroes were suffering from some cause of proportionate short stature. The fact that the seven dwarfs were siblings is compatible with a similar underlying genetic defect. Whether this disorder was transmitted from their parents through a dominant or a recessive mode of inheritance cannot be, of course, ascertained. Therefore, congenital rather than acquired causes of growth hormone deficiency apply in this case (15). Similarly, it is also highly unlikely that the totality of seven dwarf siblings were all the product of intrauterine growth retardation accompanied by failure of catch-up (16).

Another element that may help us narrow down the large number of chronic conditions associated with short stature is the apparent physical abilities of the seven dwarfs. The fact that all of them were miners points to their ability to engage in strenuous physical activity. Consequently, clinical conditions associated with hypotonia or muscular atrophy as well as disorders in organ systems, such as the heart, liver, and lungs, could be eliminated (17). Furthermore, their clearly normal social life would not be compatible with psychosocial causes of short stature, while the fact that the majority of them were overweight or obese would rule out insufficient nutrient intake (18). Iatrogenic causes could, of course, also be ruled out as well as constitutional delay of growth and puberty granted that children with the latter condition reach normal adult height in line with their genetic potential (19).

## Description, information, and data

In any emergency alert (amber or silver), together with a recent photo of the missing person, we are briefly informed about his most exceptional characteristics so that we may form a visual image of the individual. *Doc* is the leader, wears glasses, and often mixes up his words. *Bashful* has a rosy blushy face and a small shy smile. He is very shy and sweet. *Grumpy* has the biggest nose of the dwarfs and is frequently seen with one eye shut. *Sneezy* suffers from extraordinarily powerful sneezes, which are seen blowing even the heaviest of objects across a room. *Sleepy* is always tired and appears lethargic in most situations. *Happy* is the joyous dwarf and is usually portrayed laughing, while *Dopey* is mute, clumsy, and beardless (3). Analyzing the above, we see that Doc and Dopey represent the extremes of the spectrum of a specific clinical condition (Doc the tallest and smartest, Dopey the shortest...as well as ‘dopey’). In other words, we are looking for a clinical condition which, in its full expression, substantially decreases growth and cognition, while in milder forms these two aspects are less affected. These characteristics are presented in Table 1.

**Figure 3:**
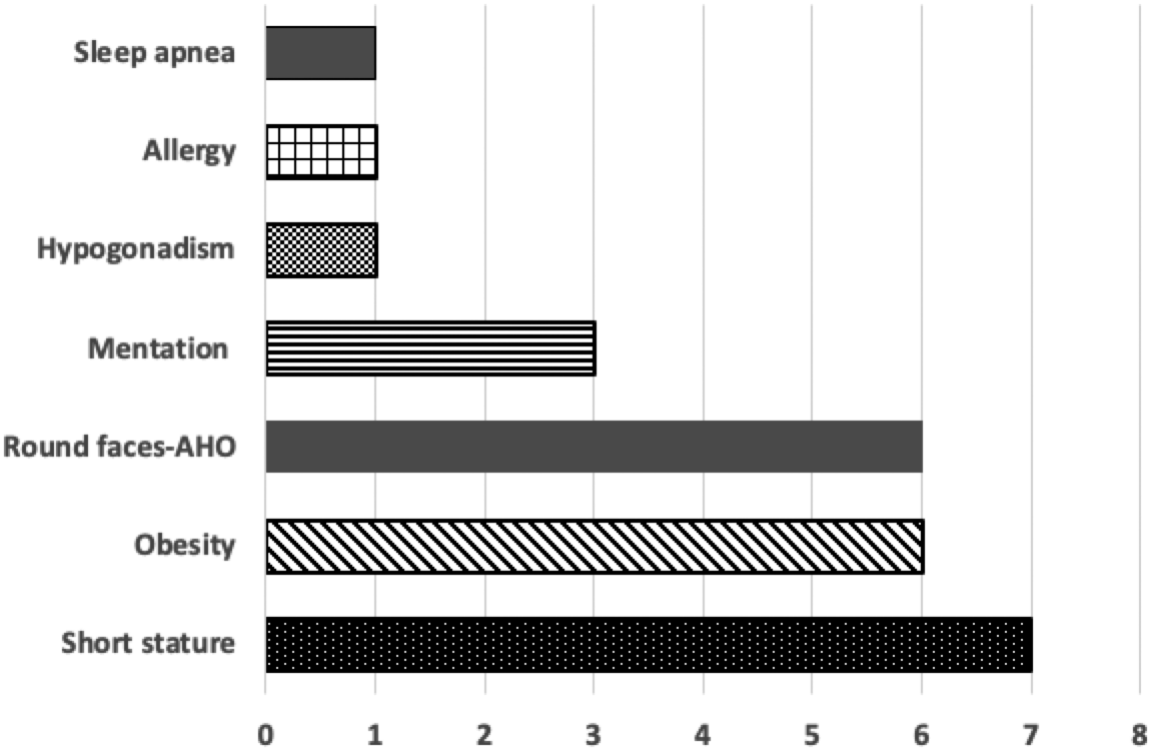
Plausible features depicted in Walt Disney’s 1937 film *Snow White and the Seven Dwarfs*.

By pooling the above information, we are able to zoom in on a range of clinical conditions that would pertain to the seven dwarfs. Thus, it seems that their intellectual ability ranged from normal to low (Doc to Dopey), while their emotional status varied from happiness to depression (Bashful, Grumpy, Happy). Most of them were obese and had chubby, round faces. Furthermore, Sneezy obviously suffered from allergies and concurrent respiratory infections and Sleepy probably from sleep apnea. Finally, Dopey’s absence of beard is compatible with hypogonadism, therefore, he most probably exemplifies the most severe form of the disorder affecting all members of this family. We are, therefore, searching for a genetic disorder with variable phenotypes causing short stature, obesity, round facies, mental retardation, sleep apnea, and hypogonadism. All these plausible features are depicted in Fig 3.

## The usual suspects

We will now focus on several endocrinopathies associated with the abovementioned clinical characteristics. Endocrine disorders compatible with these features are hypothyroidism, Cushing syndrome, hypopituitarism/GH deficiency, and pseudo-hypoparathyroidism. Although severe forms of congenital *hypothyroidism* should be ruled out due to the devastating neurodevelopmental consequences that it can bring about, we propose that the genetic basis of milder forms could be investigated (20). Thyroid dysgenesis usually occurs sporadically, with only 2–5% of cases being attributable to identifiable genetic mutations. The transcription factors PAX8, NKX2-1, and FOXE1 are all expressed in the developing thyroid and disruption of any of these genes can lead to failure of normal thyroid gland formation (21). Unlike thyroid dysgenesis, dyshormonogenesis is frequently due to a genetic defect in some element of thyroid hormone synthesis. Known genetic causes of dyshormonogenesis include mutations in thyroglobulin (TG), thyroperoxidase (TPO), dual oxidase 2 (DUOX2) and its accessory protein (DUOXA2), the sodium-iodide symporter (SLC5A5), pendrin (SLC26A4), and iodotyrosine deiodinase (22).

Central congenital hypothyroidism is caused by dysfunction of hypothalamic or pituitary control of the thyroid gland because of inadequate production or action of TSH. Specific defects in TRH or TSH signaling may result in isolated central congenital hypothyroidism. Until recently, the only known genetic causes of this condition were very rare mutations in the TRH receptor (TRHR) or the TSHβ-subunit (TSHB) (23, 24). However, a 2012 study of 11 families with central congenital hypothyroidism discovered that loss-of-function mutations in the IGSF1 gene caused an X-linked syndrome of central hypothyroidism (25). Numerous cases of IGSF1 deficiency have since been described, making it the most common identifiable genetic cause of isolated central congenital hypothyroidism (26).

In addition to central hypothyroidism, further phenotypic findings include clinical and mild neurological phenotypes in affected males, such as hypotonia, delayed psychomotor development, clumsy behavior, and attention deficit disorder (27). The apparent lack of full clinical concordance of congenital hypothyroidism may be explained by a confluence of rare variants in several genes. The observed high rate of discordance for thyroid dysgenesis among monozygotic twins (who share nearly all variants in all genes) implies that a significant proportion of congenital hypothyroidism can most likely not be explained by germline genetic changes alone (28, 29). However, the assumed hypogonadism observed in Dopey and Doc’s excellent cognitive abilities, Happy’s constant good mood, and the dwarf brothers’ ability to work in the mines cannot reasonably be explained against a background of congenital hypothyroidism.

The diagnosis of *Cushing syndrome* is intriguing. Endogenous Cushing syndrome is rare, with an incidence of 0.7–2.4 per million population per year. In childhood and adolescence, as in adults, exogenous glucocorticoid administration is the most common cause of Cushing syndrome, which definitely cannot not be the case in the Grimm Brothers’ fairytale (30). In children, adrenocorticotropic hormone (ACTH) overproduction from a pituitary adenoma (Cushing disease) accounts for approximately 75% of all cases of Cushing syndrome in children over 7 years. In children under 7 years, adrenal causes of this disorder make up roughly 15% of childhood Cushing syndrome, including bilateral hyperplasia, adenoma, or carcinoma. Ectopic ACTH and corticotropin-releasing hormone production is another rare cause of Cushing syndrome, accounting for less than 1% of cases in adolescents (31, 32). ACTH-producing pituitary adenomas are the most common cause of Cushing syndrome in childhood, and recently mutations in the ubiquitin-specific protease 8 (USP8) gene have been disclosed in a significant portion of corticotropinomas (33). Cushing disease is usually sporadic, though it may also be familial, and is known to occur in the context of MEN-1 and, rarely, due to mutations of the AIP gene (34). ACTH-secreting tumors have also been associated with mutations in cyclin E (CCNE), EGFR, CMPtk, and LAPTM4B in adults (defects that are, incidentally, rare in the pediatric Cushing disease population) (35). Primary pigmented nodular adrenocortical disease (PPNAD) is responsible for 2% of endogenous Cushing syndrome and is more frequent in females. The majority of cases are diagnosed post-pubertally, suggesting that growth is not affected in these patients. Furthermore, more than 90% of reported cases of PPNAD occur as one of the manifestations of Carney complex, which is accompanied by spotty skin pigmentation (lentigines, usually present around the lips, eyes, or genitalia) (36).

Establishing a diagnosis based on clinical features alone can be difficult. Some of the features that are thought to most reliably distinguish Cushing syndrome from simple obesity include proximal muscle weakness, easy bruising, and violaceous skin striae greater than 1 cm wide. Decreased linear growth along with progressive weight gain is one of the hallmarks of pediatric Cushing’s syndrome, while decreased bone mineral density, osteoporosis, and fractures are present in 50–80% of patients (37). Bone loss can be more severe in primary adrenal disease compared to that due to an ACTH-producing adenoma. This may be related to a protective effect of the higher adrenal androgen levels produced by increased ACTH stimulation. However, these findings have not been reproduced in many studies, and the significance of disease etiology in bone loss and fractures remains controversial (38). Psychiatric and cognitive deficits are present in 70–85% of patients with Cushing syndrome. Depression, emotional lability, and irritability are the most common manifestations, while acute psychosis, mania, anxiety, panic attacks, suicidal ideation, and paranoia are infrequently encountered in this population (39). Hypercortisolemia is also associated with a decrease in brain volume, particularly of the hippocampus, and related impairment in learning along with short-term memory deficits (40). Excess glucocorticoids have a catabolic effect on skeletal muscles, skin, and connective tissue. Increased protein wasting and type II muscle-fiber atrophy are associated with significant muscle weakness, together with predominant involvement of the pelvic girdle musculature (41). Vertebral fractures, back pain, and depression lead to decreased mobility and disuse muscle atrophy. Persistent impairment of quality of life, primarily physical, and persistent muscle weakness have been documented even several years after remission (39). All things considered, some of the clinical features seem to apply to some of our ‘subjects’. However, while obesity is obvious in all the dwarfs and irritability is manifest in Grumpy, hypomania in Happy, and learning impairment in Dopey, the rarity of familial occurrence of Cushing disease and, most importantly, the absence of muscle weakness and decreased mobility in the dwarfs make this diagnosis a less attractive candidate.

Regarding *combined pituitary hormone deficiency* (CPHD), also known as panhypopituitarism, this disorder is possibly detectable in several of the seven dwarfs’ clinical manifestations. Indeed, it is probable that the illustrators of the movie version of the Grimm Brothers’ fairytale might have seen or even studied individuals with CHPD, even though it is a rare disorder, with an estimated prevalence of 1 in 8000 individuals worldwide. The genetic basis for combined CPHD is complex, involving over 30 genes in a variety of syndromic and non-syndromic presentations. Recessive mutations within the pituitary-specific transcription factor prophet of Pit1, or PROP1, are associated with CPHD (GH, prolactin (PRL), and TSH deficiency with additional LH and FSH deficiency). Mutations within POU1F1 are associated with GH, TSH, and PRL deficiencies, with TSH deficiency being highly variable (42).

Accumulating data concerning the genetic etiology of CPHD suggest that it is part of a spectrum disorder, with holoprosencephaly and septo-optic dysplasia at the severe end and hypogonadotropic hypogonadism (HH) and isolated GH deficiency (IGHD) at the mild end of the spectrum. CPHD and HH have overlapping genetic etiologies (43). In familial cases involving multiple affected individuals, the likelihood that the cause is genetic is increased, although environmental factors could play a role in the phenotypic diversity encountered in these families. Moreover, CPHD may prove to be a multifactorial disease as well, because identical mutations may produce a spectrum of phenotypic severity in different CPHD patients and incomplete penetrance is not uncommon in family pedigrees (44). Truncal adiposity (evident in all of our study dwarfs, except Dopey) is one of the most important clinical findings of GHD and GH resistance due to reduced lipolysis by GH-sensitive lipase. This condition may be a direct effect of the lack of GH as it is not reversed by IGF-I treatment in GH insensitivity syndrome (45).

HH is characterized by gonadotropin insufficiency, reduced sex steroid production, and delayed puberty or infertility. It can be caused by reduced production of GnRH due to failed migration of GnRH neurons during development as well as by impaired regulation of GnRH secretion. Mutations in many genes can cause HH, and some of these genes are also implicated in CPHD (among them, CHD7, PROKR2, WDR11, FGFR1, and FGF8). In humans, a differentiating diagnosis between PROP1 and POU1F1 patients can be the presence or absence of gonadotropin deficiency, respectively, in addition to low GH, TSH, and PRL (46). In our ‘study group’, the candidate genes related to the phenotypic characteristics are PROP1 and SOX2, with PROP1 mutations being the most common genetic cause of CPHD. Patients with mutations in PROP1 are typically initially identified because of short stature due to GH deficiency. Most patients also exhibit reduced TSH (reflected in the various intellectual statuses of our dwarfs) and PRL at the time of diagnosis. At the onset of puberty, many patients with PROP1 mutations also exhibit LH and FSH deficiency and fail to develop secondary sexual characteristics (possibly applicable to Dopey). The loss of gonadotropins may also present as an evolving characteristic identified in adulthood. Although pituitary ACTH secretion should not be affected in patients carrying PROP1 mutations, several cases of secondary adrenal insufficiency have been reported, probably due to the loss of paracrine input from the other pituitary cells (47, 48).

With regard to SOX2, the clinical features associated with SOX2 mutations include eye disorders (anophthalmia and microphthalmia), brain malformations (usually hippocampal), esophageal atresia, genital abnormalities, hypoplastic anterior pituitary and intellectual disabilities. Pituitary masses have been observed in affected patients, but they do not progress. The hypothalamic-pituitary phenotypes caused by variants in SOX2 include HH or CPHD (49). A combination of intellectual disabilities and HH could be reflected in Dopey’s clinical phenotype. On the other hand, the other symptoms of the disease do not seem applicable to the seven dwarfs. Finally, a rare cause of familial short stature is gene variants, including IGF-1R heterozygous mutations or haploinsufficiency of the IGF-1 receptor gene. However, in these patients, clinodactyly, micrognathia, and a high-arched palate are observed, accompanied by severe hypoglycemic episodes (50). These features are not compatible with our case.

## The verdict

In 1942, 5 years after the creation of the movie on which our investigation is mostly based, the renowned American endocrinologist Fuller Albright introduced the term ‘pseudohypoparathyroidism’ (PHP) to describe subjects with PTH-resistant hypocalcemia and hyperphosphatemia accompanied by a constellation of developmental and skeletal defects termed ‘Albright hereditary osteodystrophy’ (AHO). These features included short stature, round facies, brachydactyly (shortening of III, IV, and V metacarpals and I distal phalanx), obesity, and soft-tissue calcifications (51). Further research revealed that PHP encompasses a heterogeneous group of endocrine disorders, namely, PHP type 1A (PHP1A), PHP type 1B (PHP1B), PHP type 1c (PHP1C), and PHP type 2 (PHP2), all characterized by normal renal function and PTH resistance as well as variable resistance to other hormones. In contrast, presence of AHO features without evidence of resistance to hormone action is designated by the term ‘pseudo-pseudohypoparathyroidism’ (PPHP) (52).

In light of the above, our working hypothesis is, thus, that our heroes suffered from PHP and, based on the features presented (4), we will attempt to provide corroboration of our hypothesis. First of all, regarding short stature, the vast majority (50–80%) of patients with PHP1A develop GHRH resistance, which subsequently leads to GH deficiency. Growth is, therefore, considerably affected and, during the prepubertal period, mild growth impairment is observed, followed by a blunted growth spurt and premature cessation of growth, resulting in short stature. Furthermore, defects in growth plates significantly contribute to the diminutive final height, as observed in all the little guys (53). Meanwhile, a big nose and round face are observed in 85% of the original Disney production drawings of the dwarfs, these characteristics being compatible with AHO, even in the absence of brachydactyly, which is a fairly common sign of AHO. However, absence of the latter feature may simply be attributable to Disney’s desire to depict the seven dwarfs with a more ‘normal’ appearance.

As it concerns obesity, which is evident in six of the seven ‘subjects’, that is, excepting Dopey, it is of note that children with PHP1A show decreased resting energy expenditure accompanied by hyperphagic symptoms (54). However, during adolescence and early adulthood, energy expenditure is enhanced, whereas hyperphagia declines and, consequently, obesity is less pronounced in adulthood than in childhood (55). Although sleep apnea is a well-known complication of obesity, the observation that sleep disturbances are more often encountered (4.4-fold higher risk) in patients with PHP1A than in control subjects with comparable BMI is particularly intriguing (56, 57). This highly unusual feature exactly tallies with Sleepy’s behavior.

Since three dwarfs (Bashful, Dopey, and Grumpy) suffered from mild-to-moderate cognitive dysfunction (Fig 3), this strongly indicates the diagnosis of PHP1A, given that mentation is impaired in approximately half of these patients and, moreover, subjects who have developed intracranial calcifications may experience seizures related to chronic neuropathic changes (58). With regard to Sneezy’s extraordinary bouts of sneezing, it is of interest that increased prevalence of asthma and allergies are observed in patients with PHP1A (59). Finally, these patients may be hyporesponsive to the biologic effects of other peptide hormones that use Gsα to enhance cAMP production (60). Hypogonadism due to resistance to the action of gonadotropins is highly variable and has been poorly investigated, although it appears to be a compatible diagnosis with the absence of beard, as seen in Dopey. With regards to our specific investigation, given the fact that PHP is associated with skeletal deformities, one would wonder how the seven dwarfs managed to execute the backbreaking work in the mine without suffering any fractures. However, there are several studies indicating that the bone remodeling response to PTH is unharmed in PHP, leading to what would have been normal bone metabolism and bone mineral density in these ‘subjects’, which would allow them to carry out this grueling work (61, 62).

One may logically ask why we arrived at the verdict that PHP1a must be the correct diagnosis given such a great variety of different clinical findings in the seven dwarfs. The fact is that our present-day expanding knowledge of the role of epigenetics in almost any function of the developing organism has hugely contributed to the understanding of this disorder and, thus, aided us in what we feel is the firm establishment of a diagnosis. The term ‘epigenetics’ denotes any modification of gene expression which cannot be attributed to changes in DNA sequence. PHP *per se* constitutes an epigenetic disease, since it is inherited in an autosomal dominant manner and, interestingly, the complete form (multihormone resistance with AHO) is observed only upon maternal inheritance of stimulatory G protein (Gsα) mutations, while patients inheriting the disease from the father suffer from PPHP (63, 64). Although PPHP cannot be excluded from the differential diagnosis, the lack of laboratory tests from our patients does not provide the needed material for a solid diagnosis of the above mentioned condition. However, one point against PPHP diagnosis is obesity is less prominent or absent among subjects with PPHP.

PHP1A is a result of loss of maternal expression of the GNAS gene that encodes the subunit of the Gsα mediating the signal transduction of the PTH receptor, whereas PHP1B results from methylation defects of maternally derived GNAS differentially methylated regions. However, recent data on both clinical and molecular aspects of these complex disorders have challenged the notion that there is a clear distinction between the various GNAS-related diseases, showing that some of them (PHP1A, PHP2B, and PHP1C) share more clinical and molecular similarities than originally described. Indeed, similar GNAS molecular alterations may lead to a broad spectrum of diseases, from isolated PTH resistance to multihormone resistance associated with AHO (65, 66). In addition, the expression of GNAS haploinsufficiency is tissue-specific, strengthening the selective resistance to several hormones and the observed variation of symptom severity. Moreover, analysis of PHP patients has shown the existence of different degrees of methylation defects associated with PHP1B, this possibly pointing to the presence of epigenetic mosaicism in these patients (67). On top of that, not to be overlooked, are epigenetic modifications of other hormonal/cognitive systems during the early years of life, which may affect detrimentally adult behavior and function (68).

It is well established that naturally occurring variations in maternal care during the first years of life are associated with changes in brain activity and behavior that may persist throughout adulthood. These effects are reversed by cross-fostering, demonstrating a causal link between maternal care and gene expression programming. Equally importantly, the epigenetic response to maternal care is coordinated in clusters across broad genomic areas and not only in single candidate gene promoters, this highlighting the complexity of the early factors that affect adulthood skills and abilities (69, 70).

## Conclusions

Hypothyroidism and Cushing syndrome having been excluded from our differential diagnosis, we conclude that CHPD deficiency and pseudohypoparathyroidism are the strongest candidates for an accurate clinical diagnosis of the seven dwarfs, since these conditions may account for most of their clinical characteristics. Interestingly, it seems that Sneezy plays a key role in our final diagnosis, because although GH deficiency has not been correlated with frequent infections, patients with PHP are, in fact, at increased risk of infections (71, 72). In conclusion, PHP1A accords with all of the clinical characteristics presented by each of Disney’s seven dwarfs, making it the most likely diagnosis. What is most interesting in the abovedescribed investigation, analysis, and resultant diagnosis is the fact that endocrine challenges may be discovered everywhere, even in a fairytale. Indeed, given that the science of medicine can frequently be seen through the prism of literary and artistic depiction of human nature, and granted that this is evident in every aspect of human civilization, an ‘outside the box’ approach to examining this truth is likely to uncover new pathways in the pursuit of knowledge as well as a deeper understanding of man’s evolution and development.

## Declaration of interest

The authors declare that there is no conflict of interest that could be perceived as prejudicing the impartiality of this review.

## Funding

This work did not receive any specific grant from any funding agency in the public, commercial or not-for-profit sector.
